# Biochemical Profile at Residential Admission in Anorexia Nervosa: Comparable Findings Across Restricting and Binge–Purge Subtypes

**DOI:** 10.3390/nu18162658

**Published:** 2026-08-14

**Authors:** Francesco Monaco, Annarita Vignapiano, Stefania Landi, Ernesta Panarello, Rossella Bonifacio, Daniela Falchetta, Alessandra Marenna, Domenico Di Rosa, Alessia Collura, Annarita Mainardi, Luca Steardo, Gennaro Sosto, Giulio Corrivetti

**Affiliations:** 1 Department of Mental Health, ASL Salerno, 84126 Salerno, Italy; fmonaco1980@gmail.com (F.M.); annarita.vignapiano@gmail.com (A.V.); rosbonifacio@yahoo.it (R.B.); daniela.falchetta@gmail.com (D.F.); alessiacollura97@gmail.com (A.C.); a.mainardi@aslsalerno.it (A.M.); lucasteardo@gmail.com (L.S.J.); 2 European Biomedical Research Institute of Salerno (EBRIS), 84126 Salerno, Italy; a.marenna@ebris.eu (A.M.); d.dirosa@ebris.eu (D.D.R.); corrivetti@gmail.com (G.C.); 3 Department of Psychology, University of Campania “Luigi Vanvitelli”, 81100 Caserta, Italy; ernestapanarello@gmail.com; 4 General Management, ASL Salerno, 84126 Salerno, Italy

**Keywords:** anorexia nervosa, biochemical phenotype, residential eating disorder treatment, malnutrition, low T3 syndrome, starvation hepatopathy, micronutrient deficiencies, comparable biochemical profiles across subtypes

## Abstract

**Background:** Anorexia nervosa (AN) is associated with extensive biochemical alterations at the time of clinical presentation. Despite distinct behavioural profiles, it remains unclear whether the restricting (AN-R) and binge–purge (AN-BP) subtypes differ in their biochemical profile at residential admission, a question with direct implications for clinical monitoring protocols. **Methods:** We conducted a retrospective cross-sectional study of 177 consecutive AN inpatients (AN-R: n = 130; AN-BP: n = 47; 98.3% female; mean BMI 15.8 ± 2.0 kg/m^2^; mean age 19.2 ± 5.5 years; 53.7% adolescents) admitted to a specialist residential unit (2018–2026). Thirty-five biochemical parameters were assessed. Unadjusted comparisons used Mann–Whitney U tests with rank-biserial effect sizes; multivariable logistic regression adjusted for BMI, age, and illness duration. **Results:** Overall, 98.3% of patients presented at least one biochemical anomaly (mean 4.4 ± 2.6 per patient). The most frequently abnormal parameters were LDH elevation (75.0%), vitamin D insufficiency (73.0%), low T3 syndrome (defined as low free triiodothyronine [FT3]) (58.0%), FT4 suppression (53.5%), ALT elevation (35.1%), and leucopenia (32.3%). AN subtype predicted none of these outcomes in unadjusted or multivariable analyses (all *p* > 0.10). The odds of presenting ≥3 simultaneous anomalies were not significantly associated with subtype, BMI, or illness duration in multivariable analysis. In contrast, older age independently predicted greater odds of low T3 syndrome (OR = 1.14 per year, 95% CI [1.00, 1.30], *p* = 0.045) and leucopenia (OR = 1.22 [1.06, 1.39], *p* = 0.004). No differences were observed between adolescents and adults in unadjusted comparisons. **Conclusions:** Despite their distinct behavioural profiles, no statistically significant biochemical differences were detected between AN-R and AN-BP at residential admission. These findings support routine standardised biochemical screening in all patients with anorexia nervosa admitted to residential care, regardless of behavioural subtype. Older age may identify a subgroup warranting closer endocrine and haematological monitoring.

## 1. Introduction

Anorexia nervosa (AN) is a severe psychiatric disorder clinically defined by a pattern of persistent energy intake restriction, an intense dread of weight gain, and a profoundly distorted perception of body shape and weight [1]. With a standardised mortality ratio among the highest of all psychiatric conditions [2], AN is associated with extensive medical complications affecting virtually every organ system. At the time of residential admission, these complications manifest as a complex biochemical picture, reflecting the cumulative metabolic, endocrine, and haematological consequences of chronic malnutrition, which serves both as a severity indicator and as a guide for treatment planning.

Previous studies have documented individual biochemical abnormalities in AN, including suppression of thyroid hormones [3,4,5,6,7], hepatic transaminase elevation [8,9,10,11,12], haematological changes [13,14,15], and electrolyte disturbances [16,17,18]. However, systematic characterisations of the full biochemical profile at residential admission, examining a comprehensive panel of parameters simultaneously in a large consecutive cohort, remain sparse, and existing studies are often limited to selected analytes or non-residential settings [19,20,21]. As a consequence, clinicians currently lack a comprehensive evidence base from which to construct standardised biochemical assessment protocols at admission; this may contribute to variability in test ordering and to diagnostic omissions in routine clinical practice.

A central unresolved question concerns whether the biochemical profile at admission differs between the two principal AN subtypes: the restricting subtype (AN-R) and the binge–purge subtype (AN-BP). Despite their distinct behavioural profiles, one characterised by restriction, the other by recurrent binge-eating and compensatory behaviours, it remains unclear whether the shared pathophysiological substrate of severe and chronic malnutrition is sufficient to drive convergence toward a common biochemical phenotype. Resolving this question has direct clinical implications: if the profiles are indistinguishable, a single standardised assessment protocol is appropriate for all AN patients; if they differ, subtype-specific monitoring may be warranted.

The present study addressed these gaps by providing a systematic description of 35 biochemical parameters at residential admission in 177 consecutive AN patients, and by examining whether the biochemical profile differs between AN subtypes, between adolescents and adults, and whether clinical variables independently predict specific anomalies in multivariable models.

## 2. Materials and Methods

### 2.1. Study Design and Setting

This was a retrospective cross-sectional study conducted at Regional Residential Centre for Eating Disorders “Mariconda” (Salerno, Italy). Data were collected from consecutive admissions between January 2018 and June 2026.

### 2.2. Participants

Inclusion criteria: (1) DSM-5 diagnosis of AN (restricting or binge–purge subtype) confirmed by a senior psychiatrist at admission using the Structured Clinical Interview for DSM-5 Disorders (SCID-5); (2) age ≥ 12 years; (3) routine biochemical blood panel available within 72 h of admission. Patients with co-occurring type 1 or type 2 diabetes mellitus, chronic liver disease, thyroid disorder pre-dating the AN diagnosis, or current thyroid hormone replacement therapy were excluded. A total of 177 patients met inclusion criteria and constituted the study sample (Table 1).

### 2.3. Biochemical Assessments

**Figure 1:**
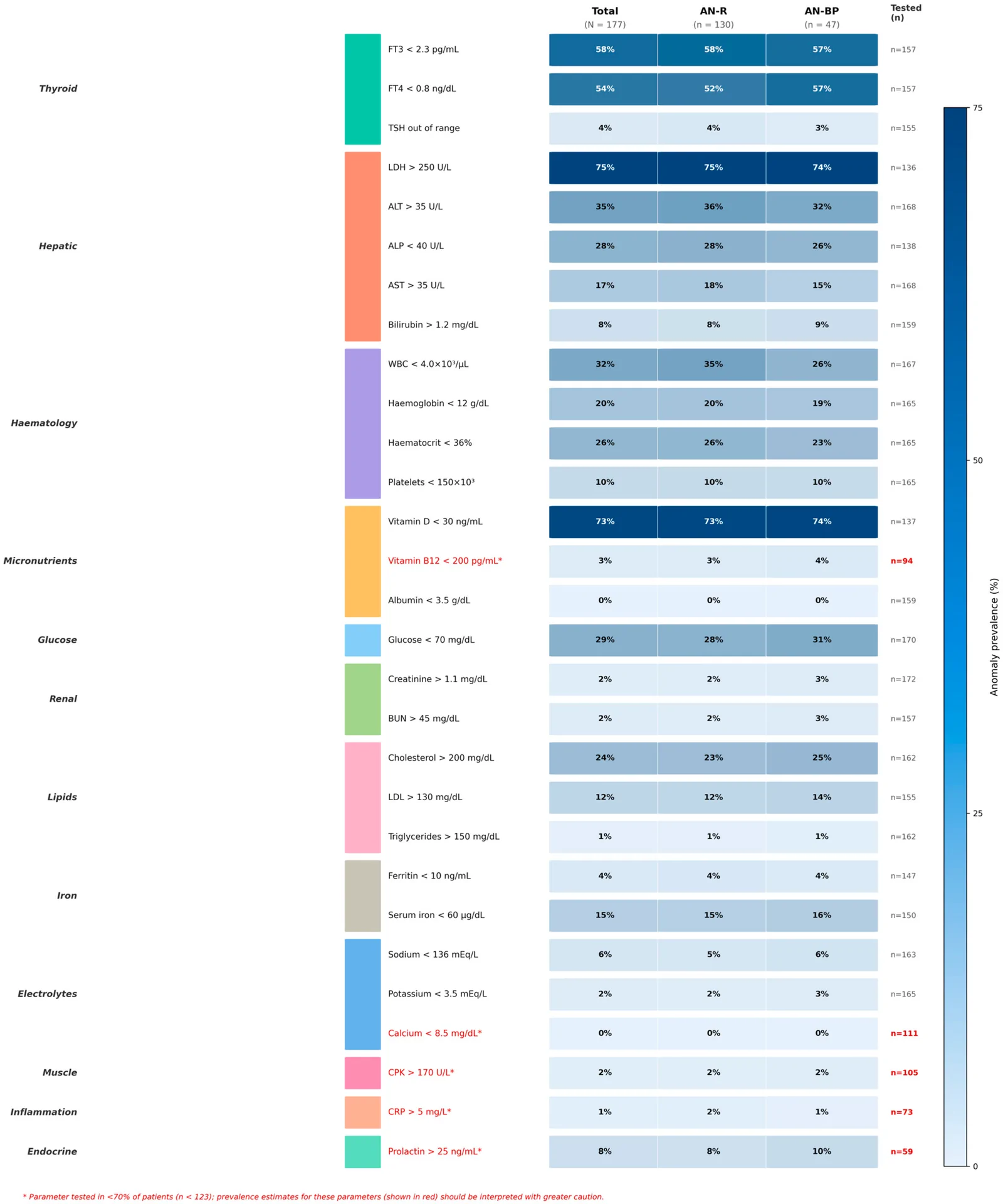
Prevalence of biochemical anomalies at residential admission in anorexia nervosa (N = 177). Note: Heatmap showing the proportion of tested patients with clinically abnormal values for 35 biochemical parameters, grouped by organ system, in the total sample and by AN subtype (AN-R, n = 130; AN-BP, n = 47). Colour intensity encodes anomaly prevalence from 0% (light blue) to ≥75% (dark blue). The right-hand column reports the number of patients tested for each parameter (n); parameters available in fewer than 70% of patients (n = 124) are marked with an asterisk (*) and highlighted in red, indicating that prevalence estimates for these parameters should be interpreted with greater caution.

Routine blood samples were drawn after overnight fasting (minimum 8 h) within 72 h of admission, prior to nutritional rehabilitation. Samples were analysed at the accredited clinical laboratory of ASL Salerno using standard automated platforms with internal and external quality control procedures. Thirty-five parameters were assessed across the following domains: haematological indices; glucose metabolism; hepatic function (ALT, AST, GGT, bilirubin, ALP, LDH); renal function; electrolytes; thyroid function (TSH, FT3, FT4); lipid profile; iron status; micronutrients (vitamin D, vitamin B12); inflammatory markers (CRP); muscle enzymes (CPK); and endocrine parameters (prolactin). Laboratory requests were made at the discretion of the admitting physician according to individual clinical presentation, reflecting routine clinical practice rather than a pre-specified research protocol. Consequently, not all biochemical parameters were available for every patient. Missingness therefore reflected routine clinical decision-making rather than selective exclusion based on patient characteristics or laboratory results. For the anomaly burden outcomes (total number of abnormalities per patient, ≥1 abnormality, ≥3 simultaneous abnormalities), a parameter was counted as abnormal only if it was tested and the result fell outside the pre-specified reference range; patients not tested for a given parameter were excluded from the denominator for that parameter, so the denominator varies by parameter as shown in Table 2 and Figure 1. Complete case analysis was performed separately for each parameter, and the corresponding sample size is reported throughout. Patients with missing data did not differ systematically from those with available data in terms of BMI or illness duration.

### 2.4. Reference Ranges and Anomaly Definition

Clinically relevant thresholds were defined a priori based on standard laboratory reference ranges and published guidelines for medical complications of eating disorders [20]: anaemia (haemoglobin < 12.0 g/dL in females, per WHO criteria), leucopenia (WBC < 4.0 × 10^3^/µL), hypoglycaemia (fasting glucose < 70 mg/dL), transaminase elevation (ALT or AST > 35 U/L), low ALP (<40 U/L), LDH elevation (>250 U/L), low T3 syndrome (defined here as low FT3 < 2.3 pg/mL with normal TSH [3,7]), vitamin D insufficiency (<30 ng/mL, per Endocrine Society criteria), and hypokalaemia (potassium < 3.5 mEq/L). The TSH reference range was 0.4–4.0 mIU/L. For alkaline phosphatase (ALP), the lower limit of normal of 40 U/L is the adult reference range of the institutional laboratory, applied uniformly to all patients. In adolescent patients, physiologically elevated bone-derived ALP isoenzyme activity during active skeletal growth raises the normal lower limit above adult values; the adult threshold therefore provides a conservative (likely underestimated) prevalence figure for ALP insufficiency in the adolescent subgroup.

### 2.5. Statistical Analysis

Continuous variables are described as median and interquartile range (IQR). Group comparisons between AN-R and AN-BP, and between adolescents (<18 years) and adults (≥18 years), used Mann–Whitney U tests; effect sizes were estimated as rank-biserial correlation (r). Inter-parameter Spearman correlations were computed for ten key variables. Multivariable binary logistic regression was performed for seven pre-specified outcomes, comprising six individual anomalies and a composite outcome of ≥3 simultaneous anomalies, each adjusted for AN subtype, BMI, age, and illness duration (covariates selected a priori based on clinical relevance). To firmly assess potential multicollinearity between age and illness duration (ρ = 0.69), Variance Inflation Factors (VIFs) were calculated for all multivariable models. All VIF values were strictly <2.5 (range 1.03–1.43), confirming the mathematical stability and validity of the simultaneous covariate adjustment. A *p*-value < 0.05 was considered statistically significant. A sensitivity analysis for the composite outcome (number of simultaneous anomalies ≥ 3) was performed restricting the count to the 14 biochemical parameters available in ≥85% of the cohort (haemoglobin, WBC, glucose, ALT, AST, potassium, sodium, FT3, FT4, TSH, creatinine, albumin, total cholesterol, and haematocrit; minimum n = 155, 88%), ensuring a consistent denominator across patients. No correction for multiple comparisons was applied to exploratory comparisons, consistent with the descriptive primary aim of the study. Given the exploratory nature of secondary analyses, findings should be interpreted accordingly and require confirmation in independent cohorts. Analyses were performed using Python 3.11 (SciPy 1.11).

### 2.6. Ethical Statement

The study was conducted in accordance with the Declaration of Helsinki. Patient consent was waived due to the retrospective design and fully anonymised data, in accordance with Italian Legislative Decree No. 196/2003 and GDPR (EU) 2016/679.

## 3. Results

### 3.1. Sample Characteristics

The study sample comprised 177 consecutive AN patients (AN-R: n = 130, 73.4%; AN-BP: n = 47, 26.6%; 98.3% female; mean age 19.2 ± 5.5 years; 53.7% adolescents; mean BMI 15.8 ± 2.0 kg/m^2^; 58.8% with BMI < 16.0 kg/m^2^; mean illness duration 3.8 ± 4.2 years). AN-R and AN-BP did not differ significantly on any demographic variable (all *p* > 0.05). Sample characteristics are presented in Table 1.

### 3.2. Prevalence of Biochemical Anomalies and Anomaly Burden

**Figure 2:**
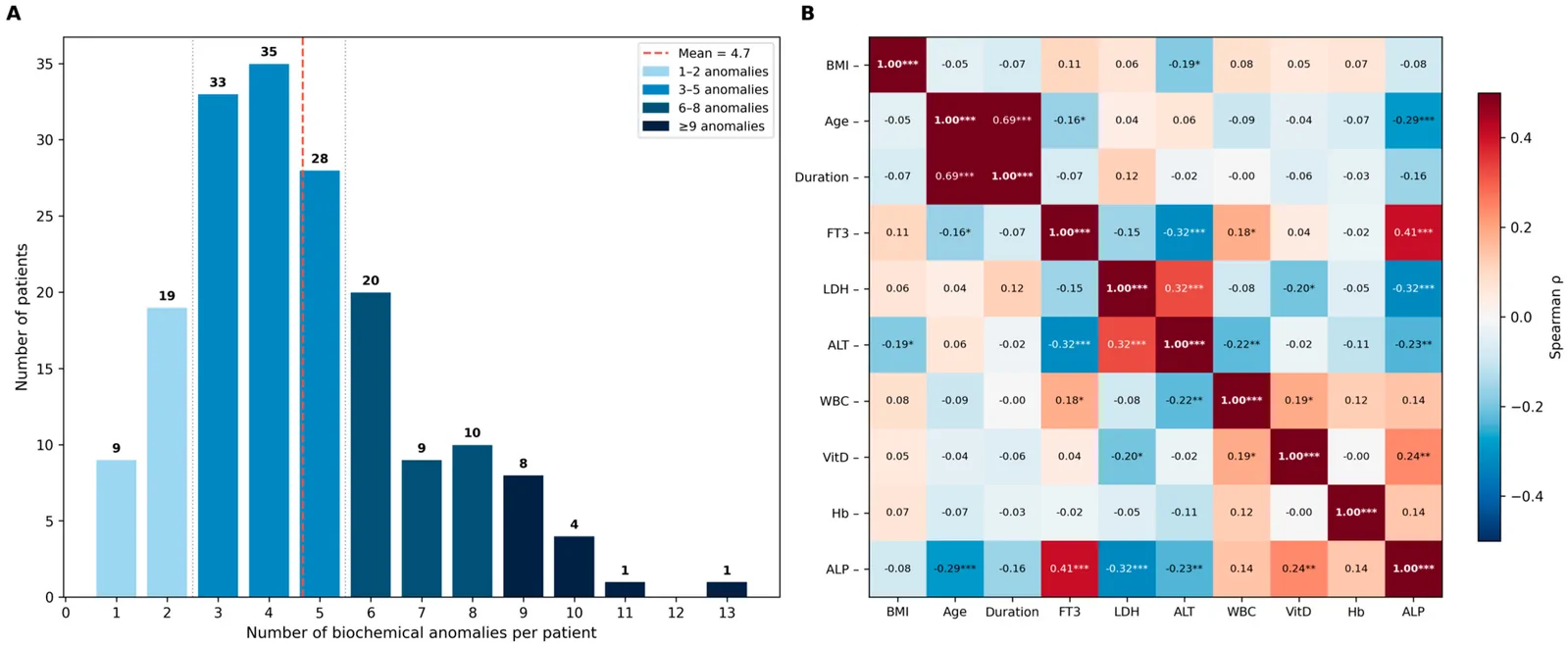
Distribution of biochemical anomalies and inter-parameter correlations. Note: (**A**) Distribution of the number of simultaneous biochemical anomalies per patient at residential admission (N = 177; mean 4.4 ± 2.6, indicated by the red dashed vertical line). AN-R and AN-BP did not differ significantly (4.5 vs. 4.2 anomalies per patient, *p* = 0.516). (**B**) Spearman correlation matrix for ten key biochemical and clinical variables. Colour encodes correlation direction and strength (red = positive, blue = negative). * *p* < 0.05; ** *p* < 0.01; *** *p* < 0.001. ALP, alkaline phosphatase; ALT, alanine aminotransferase; BMI, body mass index; FT3, free triiodothyronine; Hb, haemoglobin; LDH, lactate dehydrogenase; VitD, vitamin D; WBC, white blood cells.

Overall, 98.3% of patients (n = 174) presented at least one biochemical anomaly at admission. The number of simultaneous anomalies per patient followed an approximately normal distribution with a mean of 4.4 ± 2.6 (median 4, IQR [2–6]; Figure 2A). Only three patients (1.7%) showed a completely normal profile. A total of 73.4% (n = 130) presented three or more simultaneous anomalies, and 58.2% (n = 103) presented four or more. The most frequently abnormal parameters, in descending order, were: LDH elevation (75.0%), vitamin D insufficiency (73.0%), low T3 syndrome (58.0%), FT4 suppression (53.5%), ALT elevation (35.1%), leucopenia (32.3%), hypoglycaemia (28.8%), and low ALP (27.5%). Severe electrolyte disturbances were rare: hypokalaemia affected 2.4% of patients and hyponatraemia 5.5%. Albumin was normal in all tested patients. The complete biochemical profile for all 35 parameters is presented in Table 2 and Figure 1.

### 3.3. Comparison Between AN-R and AN-BP: Absence of Significant Biochemical Differences

No statistically significant difference was observed between AN-R and AN-BP patients on any of the 35 biochemical parameters (all *p* > 0.05). Effect sizes were uniformly negligible to small (rank-biserial r ranging from 0.013 for ALT to 0.180 for WBC). Median values were virtually identical across subtypes for all key parameters: FT3 (2.2 vs. 2.1 pg/mL), LDH (326 vs. 325 U/L), ALT (26 vs. 24 U/L), and vitamin D (22.4 vs. 23.4 ng/mL). Notably, the mean number of simultaneous anomalies per patient was 4.5 (AN-R) vs. 4.2 (AN-BP) (*p* = 0.516).

### 3.4. Comparison Between Adolescents and Adults

No significant differences were observed between adolescents (<18 years, n = 95) and adults (≥18 years, n = 82) on any biochemical parameter (all *p* > 0.05), including FT3 (2.2 vs. 2.1 pg/mL, *p* = 0.217), LDH (328 vs. 324 U/L, *p* = 0.922), ALT (24 vs. 28 U/L, *p* = 0.978), and vitamin D (22.8 vs. 22.4 ng/mL, *p* = 0.803).

### 3.5. Inter-Parameter Correlations and Multivariable Predictors

The Spearman correlation matrix (Figure 2B) revealed several biologically meaningful associations. FT3 correlated positively with ALP (ρ = 0.41, *p* < 0.001), both markers of enzymatic activity suppressed in severe malnutrition, and negatively with ALT (ρ = −0.32, *p* < 0.001). LDH correlated positively with ALT (ρ = 0.32, *p* < 0.001), consistent with shared cellular stress mechanisms. Age correlated negatively with ALP (ρ = −0.29, *p* < 0.001), suggesting progressive enzymatic suppression with increasing age in this population. BMI showed only a weak negative association with ALT (ρ = −0.19, *p* < 0.05) and no significant correlation with thyroid parameters. However, the fact that serum albumin remained completely normal in 100% of tested patients suggests a classic marasmic adaptation pattern. In this state, hepatic protein synthesis is relatively preserved while skeletal muscle structural proteins are heavily catabolized for energy salvage, a multi-compartmental tissue wasting that likely drives the massive systemic release of LDH.

**Figure 3:**
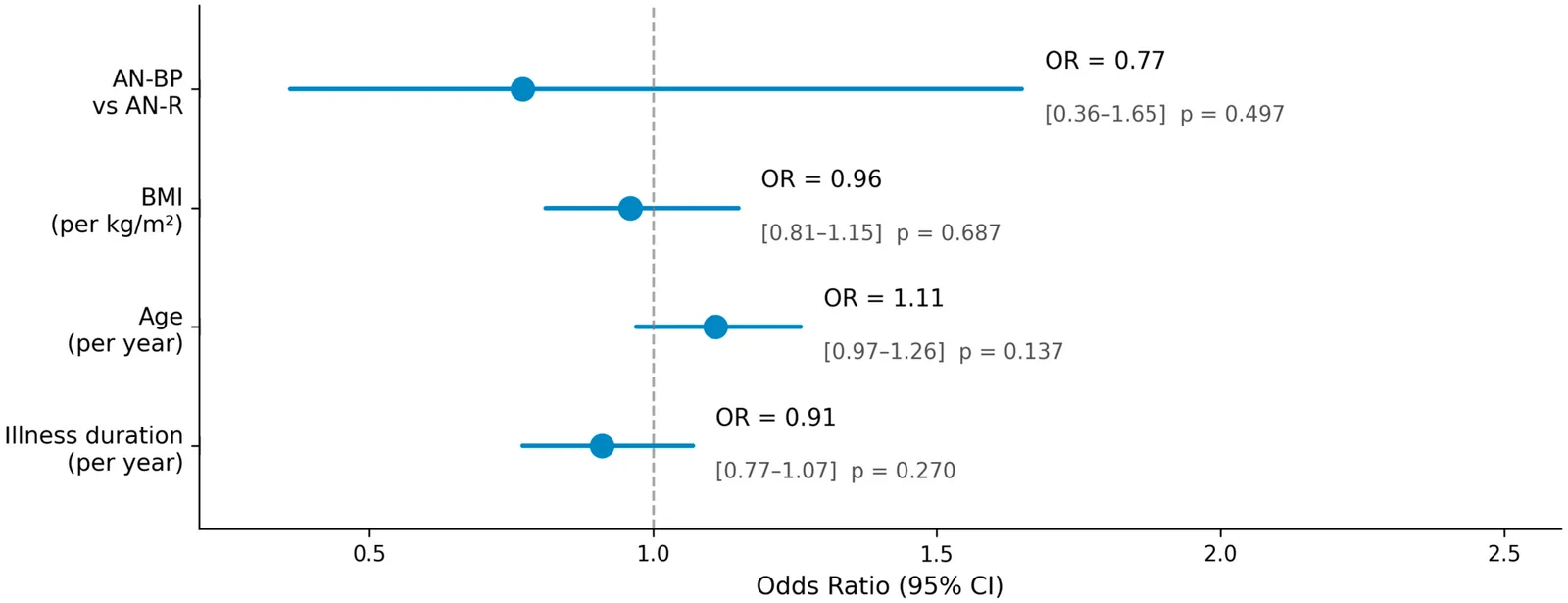
Multivariable predictors of presenting with ≥3 simultaneous biochemical anomalies at residential admission in anorexia nervosa. Note: Forest plot of odds ratios (ORs) and 95% confidence intervals from a binary logistic regression model adjusted simultaneously for AN subtype, BMI, age, and illness duration. Model statistics: N = 177; events (≥3 anomalies) = 130 (73.4%); C-statistic = 0.586; Nagelkerke R^2^ = 0.023; all VIF values < 2.5 (range 1.03–1.43). No predictor reached statistical significance, indicating that a high biochemical burden showed no significant differences in distribution across clinical subgroups according to AN subtype, BMI, age, or illness duration.

In multivariable logistic regression models (Table 3), AN subtype was not a significant predictor of any biochemical outcome (all *p* > 0.10 for AN-BP vs. AN-R). BMI was not a significant predictor in any model (all *p* > 0.05). Two independent predictors emerged: older age was independently associated with greater odds of low T3 syndrome (OR = 1.14 per year, 95% CI [1.00, 1.30], *p* = 0.045) and leucopenia (OR = 1.22, 95% CI [1.06, 1.39], *p* = 0.004). Longer illness duration was inversely associated with leucopenia (OR = 0.77, 95% CI [0.64, 0.92], *p* = 0.004), an association that may reflect survivorship or haematopoietic adaptation and should be interpreted with caution. For the composite outcome of ≥3 simultaneous anomalies (present in 73.4% of patients), no predictor reached significance (Figure 3). In a pre-specified sensitivity analysis restricting the composite count to the 14 parameters available in ≥85% of the cohort, the proportion with ≥3 simultaneous anomalies was 51.4% overall (AN-R 53.1% [69/130] vs. AN-BP 46.8% [22/47]; Mann–Whitney *p* = 0.362, rank-biserial r = −0.089), confirming that the absence of a significant between-subtype difference is robust to differential test availability.

## 4. Discussion

This study characterises the biochemical profile at residential admission in one of the largest single-centre consecutive residential cohorts reported to date in anorexia nervosa, examining 35 biochemical parameters in 177 patients over an eight-year period. Although the very high prevalence of biochemical abnormalities (98.3%) confirms the profound systemic impact of severe malnutrition, the principal finding extends beyond the overall burden of abnormalities. Rather than representing a negative comparison between anorexia nervosa subtypes, the present findings show that, despite marked behavioural differences, AN-R and AN-BP showed no significant differences in any of the 35 measured biochemical parameters at residential admission. Effect sizes were negligible throughout (rank-biserial r range 0.01–0.18), and 95% confidence intervals for between-group differences in all regression models were wide and compatible with both clinical equivalence and clinically meaningful differences that this sample lacked power to detect. The absence of statistically significant differences across thyroid function, hepatic markers, haematological indices, lipid profile, and micronutrient status is consistent with the hypothesis that once severe malnutrition is established, its metabolic consequences may largely override behavioural subtype in shaping the biochemical profile at presentation, though formal equivalence has not been established. These findings do not support the existence of subtype-specific biochemical profiles at residential admission and provide a rationale for evaluating a unified, subtype-independent approach to biochemical assessment.

### 4.1. Energy Metabolism: LDH, FT3, and ALT as Markers of Cellular Stress

The most prevalent anomaly was LDH elevation (75.0%), substantially exceeding rates reported in acute inpatient cohorts [16,22]. In the residential setting, where patients have typically sustained severe malnutrition for months to years, LDH elevation likely reflects a multi-compartmental pattern of cellular stress rather than isolated organ damage. Three complementary mechanisms may contribute to LDH elevation, consistent with prior biochemical and histological evidence: starvation-induced hepatocellular autophagy [8,10,12], skeletal muscle protein catabolism [13], and subclinical haemolysis. As glucose and lipid availability declines, amino acid mobilisation from muscle and liver becomes an essential energy source, resulting in increased LDH release as part of the metabolic adaptation to prolonged starvation [10,23].

The positive correlation between LDH and ALT (ρ = 0.32, *p* < 0.001) observed in this cohort supports this multi-organ interpretation.

It should be noted that LDH isoenzyme analysis was not performed in this cohort; the relative contributions of hepatic (LDH-5), muscular (LDH-5), and haemolytic (LDH-1/LDH-2) sources to the observed LDH elevation therefore cannot be disaggregated from the available data. The mechanistic interpretation presented above should be understood as a working hypothesis consistent with the clinical context and prior literature, not as a directly demonstrated mechanism in this cohort.

Thyroid axis suppression was equally prevalent: 58.0% of patients met criteria for low T3 syndrome and 53.5% showed FT4 suppression. The positive correlation between FT3 and ALP (ρ = 0.41, *p* < 0.001), both markers of enzymatic activity that are jointly suppressed in severe malnutrition, underscores the systemic nature of this metabolic adaptation. The co-suppression of FT4 in over half of patients suggests that thyroid adaptation extends beyond peripheral deiodinase inhibition to involve central hypothalamic–pituitary–thyroid downregulation [6]. In multivariable analysis, older age but not BMI independently predicted thyroid suppression (OR = 1.14 per year, *p* = 0.045), suggesting that BMI alone may inadequately reflect the degree of metabolic adaptation once severe malnutrition has been established. ALT elevation (35.1%) is consistent with prior reports of starvation-related hepatopathy [8,9,10,24], with the most plausible mechanism, based on prior autopsy and biopsy data, being starvation-induced hepatocyte autophagy rather than inflammatory or toxic injury. Notably, serum albumin was normal in all tested patients despite severe and chronic undernutrition. This finding is consistent with the marasmatic pattern typical of anorexia nervosa, in which hepatic albumin synthesis appears relatively preserved despite marked depletion of skeletal muscle protein stores, consistent with prior observations [22,23,24,25]. Consequently, normal albumin concentrations should not be interpreted as evidence against clinically significant protein–energy malnutrition. The interpretation of preserved albumin as reflecting a marasmic-type metabolic adaptation should be regarded as a working hypothesis; formal confirmation would require longitudinal body composition data and protein metabolic flux measurements not available in this retrospective cohort.

### 4.2. Endocrine Adaptation: Vitamin D and the Micronutrient Burden

Vitamin D insufficiency was present in 73.0% of patients, a prevalence consistent with previous reports [26,27,28]. This finding probably reflects the combined effects of inadequate dietary intake, reduced sunlight exposure, impaired hepatic vitamin D metabolism associated with starvation hepatopathy, and depletion of adipose tissue stores. The positive correlation between vitamin D and WBC (ρ = 0.19, *p* < 0.05) may reflect the immunomodulatory role of vitamin D in haematopoietic function [14]. ALP reduction (<40 U/L in 27.5% of patients), which correlates positively with vitamin D (ρ = 0.24, *p* < 0.01) and FT3 (ρ = 0.41, *p* < 0.001), likely reflects the compound effect of zinc deficiency, thyroid suppression, and reduced bone turnover, all characteristic of severe and prolonged energy restriction.

### 4.3. Haematological Compromise: Leucopenia and the Bone Marrow

Leucopenia affected 32.3% of patients and was one of only two outcomes for which a significant independent predictor emerged in multivariable analysis: older age (OR = 1.22 per year, *p* = 0.004). This may reflect cumulative haematopoietic vulnerability to starvation-induced bone marrow suppression and gelatinous marrow transformation [13,14,29] in older patients with longer exposure to AN pathophysiology. It should be noted that age and illness duration showed moderate collinearity (ρ = 0.69), so the independent effect of age may partially incorporate the cumulative impact of disease chronicity; these two effects are not fully separable in the present cross-sectional design. The inverse association between illness duration and leucopenia (OR = 0.77, *p* = 0.004) was unexpected and should be interpreted cautiously. The inverse association between longer illness duration and leucopenia (OR = 0.77, *p* = 0.004) is counterintuitive and warrants careful mechanistic consideration. Two non-mutually exclusive hypotheses may account for this finding. First, a survivorship selection mechanism: patients who develop severe acute bone marrow suppression early in their illness are at disproportionate risk of fatal septic complications; those who survive into the chronic illness phase may constitute a biologically selected subgroup with greater haematopoietic resilience at a given BMI. Second, a compensatory haematopoietic adaptation mechanism: prolonged chronic undernutrition may induce a stable hypoproliferative but not overtly leucopenic haematopoietic equilibrium, differing qualitatively from the acute leucopenic responses seen early in disease evolution. Both hypotheses remain speculative in the absence of prospective longitudinal data. Furthermore, the partial collinearity between age (positive predictor of leucopenia, OR = 1.22) and illness duration (rho = 0.69) means that the illness duration coefficient is estimated in the presence of a competing, opposite-direction age effect; this statistical interaction may contribute to the apparent inverse direction of the association and underscores the need for prospective confirmation.

The rarity of severe electrolyte disturbances (hypokalaemia 2.4%, hyponatraemia 5.5%) is most parsimoniously explained by the residential selection effect: patients with haemodynamically significant dysregulation requiring acute medical intervention are typically managed in acute wards before transfer. The residential population therefore represents a metabolically stable subpopulation, in whom chronic malnutrition rather than acute electrolyte decompensation is the dominant biochemical driver [16,25].

### 4.4. Convergence and Clinical Implications

The absence of any significant between-subtype difference, confirmed across both unadjusted and multivariable analyses, including for the composite ≥3 anomalies outcome, constitutes the principal finding of this study. These findings support consideration of a standardised approach to biochemical admission assessment, given the absence of detectable subtype-specific differences, though prospective validation would be needed before clinical implementation. This would simplify clinical assessment while potentially reducing unnecessary variability in laboratory testing. The proposed core panel includes routine haematological indices, electrolytes, thyroid function (TSH, FT3, FT4), hepatic markers (LDH, ALT, AST, ALP), fasting glucose, and vitamin D (Table 4). This panel captures both universally recommended admission tests and the biochemical domains most frequently affected in the present cohort.

Contextualisation with prior literature requires attention to setting. Most existing studies characterising the biochemical profile of AN patients were conducted in acute medical stabilisation units, general psychiatric wards, or outpatient clinics, where the dominant clinical picture frequently reflects acute decompensation—electrolyte dysregulation driven by recent purging, hepatic injury during early weight restoration, or transient hypoglycaemia in the context of acute caloric restriction—in which behavioural phenotype may directly drive between-subtype differences in biochemistry. In contrast, residential care patients represent a qualitatively distinct population who have typically sustained severe, chronic malnutrition over months to years, and present with a profile dominated by the cumulative metabolic consequences of prolonged undernutrition rather than by acute behavioural events. In this context, the absence of significant biochemical differences between AN-R and AN-BP at residential admission is biologically coherent: chronic starvation may impose a common metabolic trajectory that progressively decouples from the specific behaviours that initiated it. This distinction has direct clinical implications: practices of tailoring admission panels to AN subtype are not empirically supported in the residential context, and the present findings provide a rationale for a uniform, subtype-independent admission screening protocol.

Limitations of this study include the retrospective single-centre design, absence of a control group, single-time-point assessment, and unavailability of body composition and medication data. Because laboratory testing reflected routine clinical practice rather than a standardised research protocol, some biochemical parameters were available only in a subset of patients. Although patients with missing data did not differ in BMI or illness duration, the possibility of residual selection bias cannot be completely excluded. For parameters with substantially reduced sample sizes (notably prolactin, n = 59; magnesium, n = 71; CRP, n = 73), statistical power to detect subtype differences may have been insufficient; therefore, negative findings for these parameters should be interpreted with caution. Furthermore, the cross-sectional design precludes causal inference regarding the observed associations between clinical characteristics and biochemical abnormalities. Additionally, the moderate collinearity between age and illness duration (Spearman ρ = 0.69, *p* < 0.001) means that their independent effects in multivariable models should be interpreted cautiously. Residual confounding cannot be excluded, and the findings should be confirmed in prospective multicentre cohorts. It should also be noted that the discretionary ordering of extended panels may introduce a clinician-selection bias whose direction is not neutral: parameters such as CRP, magnesium, and prolactin are more likely to be requested in patients presenting with atypical or more severe clinical features, such that the tested subsets may not be representative of the full cohort. The expected direction of this bias is toward overestimation of anomaly prevalence for selectively ordered analytes; prevalence figures for these parameters should therefore be regarded as upper-bound estimates for the broader residential AN population. Furthermore, although blood sampling was protocolled prior to formal nutritional rehabilitation, the retrospective design does not allow exclusion of pre-admission intravenous fluid resuscitation or electrolyte supplementation during emergency department stays or hospital transfers, which could have transiently corrected acute electrolyte disturbances and may partly account for the low observed prevalence of hypokalaemia (2.4%).

Formal equivalence testing (e.g., two one-sided tests, TOST) was not performed; with 47 AN-BP patients the study is insufficiently powered to pre-specify a clinically meaningful equivalence margin for most outcomes. The conclusion is therefore restricted to the absence of statistically significant differences and does not constitute a demonstration of equivalence. The confidence intervals in Table 3 illustrate the precision limitations: for example, the OR for low T3 syndrome in AN-BP versus AN-R is 0.79 (95% CI 0.35–1.80), equally consistent with a halving or a near-doubling of the odds. No correction for multiple comparisons was applied to the multivariable regression analyses; with seven pre-specified models at alpha = 0.05, the probability of at least one spurious positive finding is approximately 30%. The age–T3 association (*p* = 0.045) is the only finding approaching the significance threshold and should be interpreted with caution pending external replication. Several potentially confounding variables were not available in this retrospective dataset, including: use of thyroid hormone replacement therapy, nutritional supplements, proton pump inhibitors, or laxatives at the time of blood sampling; frequency and recency of purging behaviours; recent weight trajectory preceding admission; season of blood sampling (relevant for vitamin D); and whether patients had received intravenous fluids or electrolyte repletion at referring facilities prior to transfer. The use of a uniform adult-based ALP lower limit across a mixed-age cohort may underestimate ALP insufficiency prevalence in the adolescent subgroup. Raw BMI was used as a covariate in all regression models without age- and sex-based standardisation (BMI-SDS), limiting the interpretability of BMI as a predictor in patients aged <18 years.

## 5. Conclusions

Despite their distinct behavioural profiles, AN-R and AN-BP subtypes showed no statistically significant biochemical differences at residential admission, with a high burden of anomalies predominantly involving cellular metabolism, thyroid function, and micronutrient status, not predicted by AN subtype, BMI, or illness duration in multivariable analysis. Older age independently identifies patients at greater risk of thyroid suppression and leucopenia. These findings are consistent with a rationale for considering a standardised biochemical assessment protocol at residential admission, in keeping with the programmatic framework for AN biomarker standardisation recently outlined by the WFSBP consensus [30], though prospective validation of clinical impact remains needed, and highlight the inadequacy of BMI alone as a proxy for metabolic derangement in this severely malnourished population.

## Figures and Tables

**Table 1:** Sample characteristics by AN subtype.

Characteristic	Total (N = 177)	AN-R (n = 130)	AN-BP (n = 47)
Age, years	19.2 ± 5.5	19.1 ± 5.4	19.4 ± 5.8
Adolescents (<18 years), n (%)	95 (53.7)	69 (53.1)	26 (55.3)
Sex (female), n (%)	174 (98.3)	128 (98.5)	46 (97.9)
BMI, kg/m^2^	15.8 ± 2.0	15.8 ± 1.9	15.6 ± 2.3
Illness duration, years	3.8 ± 4.2	3.7 ± 4.0	4.2 ± 4.7
Patients with ≥1 anomaly, n (%)	174 (98.3)	127 (97.7)	47 (100.0)
Patients with ≥3 anomalies, n (%)	130 (73.4)	97 (74.6)	33 (70.2)

Note: AN-R, restricting subtype; AN-BP, binge–purge subtype; BMI, body mass index. Data are mean ± SD or n (%).

**Table 2:** Complete biochemical profile at residential admission (N = 177): all 35 parameters, stratified by AN subtype.

Parameter	N (%)	Median (IQR)	Reference Range	Anomaly %	AN-R Median	AN-BP Median	*p*
***HAEMATOLOGY***							
Haemoglobin (g/dL)	165 (93)	12.8 (12.2–13.6)	≥12.0	20.0	12.8	13.0	0.451
Red blood cells (10^6^/µL)	168 (95)	4.2 (4.0–4.6)	≥3.8	13.1	4.2	4.2	0.514
Haematocrit (%)	165 (93)	37.4 (35.9–40.1)	≥36.0	25.5	37.7	37.4	0.557
White blood cells (10^3^/µL)	167 (94)	4.6 (3.8–5.7)	≥4.0	32.3	4.4	5.2	0.077
Platelets (10^3^/µL)	165 (93)	199 (168–231)	≥150	10.3	194	213	0.264
***GLUCOSE METABOLISM***							
Blood glucose (mg/dL)	170 (96)	74.5 (69.0–81.8)	70–100	28.8	74.0	76.0	0.325
***HEPATIC FUNCTION***							
ALT (U/L)	168 (95)	25.5 (16.0–45.5)	≤35	35.1	26.0	24.0	0.902
AST (U/L)	168 (95)	23.0 (19.0–30.0)	≤35	17.3	23.0	24.0	0.386
GGT (U/L)	151 (85)	15.0 (11.0–22.0)	≤35	4.6	15.0	15.0	0.739
Total bilirubin (mg/dL)	159 (90)	0.5 (0.4–0.7)	≤1.2	8.2	0.5	0.5	0.831
ALP (U/L)	138 (78)	46.5 (38.0–60.0)	40–150	27.5	48.0	45.0	0.257
LDH (U/L)	136 (77)	325.5 (252.0–389.0)	≤250	75.0	326	325	0.898
***RENAL FUNCTION***							
Creatinine (mg/dL)	172 (97)	0.7 (0.6–0.8)	≤1.1	2.3	0.7	0.7	0.178
BUN (mg/dL)	157 (89)	28.0 (24.0–33.0)	≤45	2.5	28.0	28.0	0.959
Albumin (g/dL)	159 (90)	4.5 (4.2–4.7)	≥3.5	0.0	4.5	4.5	0.423
***ELECTROLYTES***							
Sodium (mEq/L)	163 (92)	141 (139–142)	136–145	5.5	141	140	0.303
Potassium (mEq/L)	165 (93)	4.3 (4.1–4.5)	3.5–5.0	2.4	4.3	4.3	0.840
Calcium (mg/dL)	111 (63)	9.8 (9.5–10.1)	8.5–10.5	0.0	9.8	9.7	0.534
Phosphorus (mg/dL)	103 (58)	3.9 (3.6–4.3)	2.5–4.5	1.0	3.9	3.9	0.835
Magnesium (mEq/L)	71 (40)	2.0 (1.9–2.1)	1.7–2.4	0.0	2.0	2.1	0.844
***THYROID FUNCTION***							
TSH (mIU/L)	155 (88)	1.5 (1.1–2.2)	0.4–4.0	3.9	1.6	1.5	0.121
FT3 (pg/mL)	157 (89)	2.2 (1.8–2.7)	2.3–4.2	58.0	2.2	2.1	0.624
FT4 (ng/dL)	157 (89)	0.8 (0.7–0.9)	0.8–1.8	53.5	0.8	0.8	0.441
***LIPID PROFILE***							
Total cholesterol (mg/dL)	162 (92)	164 (144–197)	<200	23.5	164	165	0.865
HDL cholesterol (mg/dL)	156 (88)	61.0 (53.0–69.0)	≥40	3.2	60.0	63.0	0.329
LDL cholesterol (mg/dL)	155 (88)	90.0 (74.0–112)	<130	12.3	90.0	91.0	0.822
Triglycerides (mg/dL)	162 (92)	65.0 (53.2–83.8)	<150	1.2	65.0	68.0	0.879
***IRON STATUS***							
Ferritin (ng/mL)	147 (83)	49.7 (23.4–86.8)	10–120	4.1	50.3	42.1	0.507
Serum iron (µg/dL)	150 (85)	86.5 (68.0–102)	60–160	15.3	85.0	89.0	0.928
***VITAMINS AND MICRONUTRIENTS***							
Vitamin D (ng/mL)	137 (77)	22.5 (16.3–30.6)	≥30	73.0	22.4	23.4	0.741
Vitamin B12 (pg/mL)	94 (53)	471 (320–637)	≥200	3.2	474	439	0.600
***OTHER PARAMETERS***							
CRP (mg/L)	73 (41)	0.1 (0.0–0.3)	<5	1.4	0.1	0.2	0.290
CPK (U/L)	105 (59)	57.0 (46.0–75.0)	<170	1.9	56.0	60.0	0.493
Prolactin (ng/mL)	59 (33)	6.4 (2.6–11.3)	<25	8.5	5.6	7.5	0.167

Note: AN-R, restricting subtype; AN-BP, binge–purge subtype; ALP, alkaline phosphatase; ALT, alanine aminotransferase; AST, aspartate aminotransferase; BUN, blood urea nitrogen; CPK, creatine phosphokinase; CRP, C-reactive protein; FT3, free triiodothyronine; FT4, free thyroxine; HDL, high-density lipoprotein; IQR, interquartile range; LDH, lactate dehydrogenase; LDL, low-density lipoprotein; RBC, red blood cells; TSH, thyroid-stimulating hormone; WBC, white blood cells. Anomaly % = proportion of tested patients with values outside the stated reference range (calculated on the N indicated in column 2, not on all 177 patients). *p*-values from Mann–Whitney U test (AN-R vs. AN-BP).

**Table 3:** Multivariable logistic regression: predictors of key biochemical anomalies and multiple anomaly burden.

Outcome	Predictor	OR	95% CI	*p*	N
***Thyroid function***					
Low T3 syndrome	AN-BP vs. AN-R	1.42	[0.64, 3.13]	0.384	157
	BMI (per kg/m^2^)	0.88	[0.74, 1.05]	0.151	157
	Age (per year)	**1.14**	**[1.00, 1.30]**	0.045 *	**157**
	Illness duration	0.90	[0.77, 1.06]	0.212	157
FT4 suppression	AN-BP vs. AN-R	0.99	[0.47, 2.09]	0.978	157
	BMI (per kg/m^2^)	1.02	[0.87, 1.20]	0.803	157
	Age (per year)	1.00	[0.90, 1.12]	0.960	157
	Illness duration	1.00	[0.87, 1.16]	0.946	157
***Hepatic markers***					
LDH elevation	AN-BP vs. AN-R	0.64	[0.26, 1.55]	0.324	136
	BMI (per kg/m^2^)	1.08	[0.87, 1.34]	0.494	136
	Age (per year)	0.98	[0.84, 1.14]	0.793	136
	Illness duration	1.08	[0.89, 1.31]	0.439	136
ALT elevation	AN-BP vs. AN-R	0.84	[0.39, 1.84]	0.664	168
	BMI (per kg/m^2^)	0.87	[0.72, 1.05]	0.143	168
	Age (per year)	1.10	[0.98, 1.23]	0.120	168
	Illness duration	0.88	[0.75, 1.02]	0.095	168
***Haematology***					
Leucopenia	AN-BP vs. AN-R	0.52	[0.22, 1.22]	0.132	167
	BMI (per kg/m^2^)	0.82	[0.66, 1.02]	0.077	167
	Age (per year)	**1.22**	**[1.06, 1.39]**	0.004 **	**167**
	Illness duration	**0.77**	**[0.64, 0.92]**	0.004 **	**167**
***Micronutrients***					
Vitamin D insuff.	AN-BP vs. AN-R	1.64	[0.62, 4.33]	0.314	137
	BMI (per kg/m^2^)	1.03	[0.82, 1.29]	0.793	137
	Age (per year)	0.98	[0.85, 1.13]	0.778	137
	Illness duration	1.05	[0.88, 1.25]	0.610	137
***Multiple anomalies (≥3)***					
≥3 simultaneous anomalies	AN-BP vs. AN-R	0.77	[0.36, 1.65]	0.497	177
	BMI (per kg/m^2^)	0.97	[0.81, 1.15]	0.687	177
	Age (per year)	1.11	[0.97, 1.26]	0.137	177
	Illness duration	0.91	[0.77, 1.08]	0.270	177

Note: Each model adjusted simultaneously for AN subtype (AN-BP vs. AN-R), BMI, age, and illness duration. Bold: *p* < 0.05. * *p* < 0.05; ** *p* < 0.01. OR, odds ratio; CI, confidence interval. ALT, alanine aminotransferase; FT3, free triiodothyronine; FT4, free thyroxine; LDH, lactate dehydrogenase.

**Table 4:** Proposed minimum biochemical assessment at residential admission, informed by observed prevalence of abnormalities in this cohort.

Domain	Recommended Tests	Rationale	Prevalence in Cohort
**Thyroid function**	*TSH*, *FT3*, *FT4*	Low T3 syndrome and FT4 suppression each exceed 50% prevalence; both reflect starvation-induced non-thyroidal illness syndrome and, in severe cases, central thyroid suppression.	FT3 ↓ 58.0% FT4 ↓ 53.5% TSH abnormal: 3.9%
**Hepatic markers**	*LDH*, *ALT*, *AST*, *ALP*	LDH is the most frequently abnormal parameter (75%); reflects multi-compartmental cellular stress including hepatic autophagy, skeletal muscle catabolism, and haemolysis. ALT/AST assess transaminase elevation; low ALP indicates metabolic and enzymatic suppression.	LDH ↑ 75.0% ALT ↑ 35.1% AST ↑ 17.3% ALP ↓ 27.5%
**Micronutrients**	*Vitamin D (25-OH)*	Vitamin D insufficiency is present in 73% of patients, reflecting restricted dietary intake, reduced sun exposure, starvation hepatopathy, and depleted adipose stores.	Vitamin D ↓ 73.0%
**Haematology**	*Full blood count* *(Hb*, *WBC*, *platelets)*	Leucopenia (32%) and anaemia (20%) reflect starvation-induced bone marrow suppression and gelatinous marrow transformation. Older age is an independent predictor of leucopenia.	WBC ↓ 32.3% Hb ↓ 20.0% Hct ↓ 25.5%
**Glucose metabolism**	*Fasting blood glucose*	Fasting hypoglycaemia affects 28.8% of patients and poses a risk of acute decompensation, particularly at initiation of nutritional rehabilitation.	Glucose ↓ 28.8%
**Electrolytes**	*Sodium*, *potassium*, *calcium*, *phosphorus*	Severe electrolyte disturbances are uncommon at residential admission (hypokalaemia 2.4%), reflecting prior stabilisation. Monitoring is essential before refeeding to detect refeeding syndrome risk.	K^+^ ↓ 2.4% Na^+^ ↓ 5.5% (Monitor pre-refeeding)
**Renal function**	*Creatinine*, *BUN*, *albumin*	Severe renal impairment is rare. Albumin is normal in all tested patients, consistent with marasmic-type protein adaptation; it does not exclude significant protein–energy depletion.	Creatinine ↑ 2.3% Albumin: 0.0%
**Iron status**	*Ferritin*, *serum iron*	Iron deficiency is present in a minority; ferritin may be elevated as an acute-phase reactant masking true depletion in the context of inflammation.	Serum iron ↓ 15.3% Ferritin ↓ 4.1%
**Extended panel** **(as clinically indicated)**	*Phosphorus*, *magnesium*, *Vit B12*, *lipid profile*, *CRP*, *CPK*, *prolactin*	Parameters with lower anomaly prevalence and variable availability in this cohort (33–63% of patients tested). Ordered according to individual clinical presentation, nutritional risk, and institutional protocols. Particularly relevant in patients with purging behaviours (electrolytes), severe malnutrition (phosphorus), or endocrine concerns (prolactin).	Availability: 33–63% of cohort (see Table 2)

Note: ↑, above reference range; ↓, below reference range. Suggested minimum biochemical panel for all patients admitted to residential treatment, irrespective of anorexia nervosa subtype. Additional laboratory investigations (e.g., phosphate, magnesium, iron status, lipid profile, inflammatory markers, vitamin B12, prolactin, and other endocrine or nutritional biomarkers) may complement the core admission panel according to the individual clinical presentation, nutritional status, and local institutional protocols.

## Data Availability

Data are available from the corresponding author (S.L.) upon reasonable request and are not publicly available.
